# Prevalence of anemia among Indigenous children in Latin America: a systematic review

**DOI:** 10.11606/s1518-8787.2022056004360

**Published:** 2022-11-18

**Authors:** Carlos Rosas-Jiménez, Engin Tercan, Olaf Horstick, Ekeoma Igboegwu, Peter Dambach, Valérie R. Louis, Volker Winkler, Andreas Deckert

**Affiliations:** I Heidelberg University Heidelberg Institute for Global Health Heidelberg Baden-Württemberg Germany Heidelberg University. Heidelberg Institute for Global Health. Heidelberg, Baden-Württemberg, Germany

**Keywords:** Indigenous, South American, Child, Anemia, epidemiology, Risk Factors, Malnutrition, Poverty

## Abstract

**OBJECTIVE:**

To describe the prevalence pattern of anemia among Indigenous children in Latin America.

**METHODS:**

PRISMA guidelines were followed. Records were identified from the databases PubMed, Google Scholar, and Lilacs by two independent researchers between May and June 2021. Studies were included if the following criteria were met: a) studied Indigenous people b) was about children (from 0 to 12 years old); c) reported a prevalence estimate of anemia; d) had been conducted in any of the countries of Latin America; e) was published either in English, Portuguese, or Spanish; f) is a peer-reviewed article; and g) was published at any date.

**RESULTS:**

Out of 2,401 unique records retrieved, 42 articles met the inclusion criteria. A total of 39 different Indigenous communities were analyzed in the articles, and in 21 of them (54.0%) child anemia was a severe public health problem (prevalence ≥ 40%). Those communities were the Aymara (Bolivia); Aruak, Guaraní, Kamaiurá, Karapotó, Karibe, Kaxinanuá, Ma-cro-Jê, Suruí, Terena, Xavante (Brazil); Cabécar (Costa Rica), Achuar, Aguaruna, Awajún, Urarina, Yomybato (Peru); Piaroa and Yucpa (Venezuela); and Quechua (Peru and Bolivia). Children below two years had the highest prevalence of anemia (between 16.2% and 86.1%). Among Indigenous people, risk factors for anemia include nutrition, poor living conditions, access to health services, racism, and discrimination. Bolivia and Guatemala are scarcely studied, despite having the highest proportion of Indigenous communities in Latin America.

**CONCLUSIONS:**

Anemia constitutes a poorly documented public health problem among Indigenous children in 21 Indigenous communities in Bolivia, Brazil, Colombia, Costa Rica, Ecuador, Guatemala, Mexico, and Peru. In all Indigenous communities included in this study child anemia was an issue, especially in younger children.

## INTRODUCTION

Anemia is a disorder in which the number of red blood cells is insufficient to meet the body's needs^1^. Iron deficiency is the most common cause of anemia, but other nutritional deficiencies, acute and chronic inflammation, parasites, and inherited or acquired diseases that affect hemoglobin synthesis and red blood cell production or survival can cause anemia^1^. It is the most common blood disorder in developing countries^2^ and the health condition that affected the greatest number of people around the world (2.36 billion people) in 2015^3^. Multiple factors often contribute to the etiology of anemia, and sociodemographic conditions play a key role, especially in low-income countries^4^. For example, Leite et al.^5^, who studied Indigenous children in Brazil, documented higher risk of anemia for boys, children with lower maternal schooling, lower household socioeconomic status, poorer sanitary conditions, presence of maternal anemia, and anthropometric deficits.

Numerous factors, ranging from a lack of accurate and easily accessible information to the very nature of Indigenous identities in Latin America and the Caribbean, hinder the determination of the exact number of Indigenous people in that region^6^. To define Indigenous peoples in an efficient manner, the International Labor Organization (ILO) Convention 169 on Indigenous and Tribal Peoples in Independent Countries provides a definition that can be used to identify at least four dimensions within Indigenous peoples: recognition of identity, common origin, territoriality, and the linguistic and cultural dimension, which must be considered when establishing operational criteria^7^. Around 58 million Indigenous people live in Latin America, constituting 9.8% of the population^8^. The proportion of the population considered Indigenous in Latin America varies by country and ranges from 41.0% in Bolivia and Guatemala, to 0.5% in Brazil, and 0% in Uruguay^6^. In many countries of the region, Indigenous children are in a highly vulnerable situation, due to very high infant mortality rates, alarming levels of malnutrition in the context of food insecurity, precarious access to water, and high prevalence of diarrheal infections^9^. The situation has become a humanitarian crisis recognized by several national governments and this illustrates the dire state of Indigenous populations in many countries of Latin America. Until now, few studies from Latin America have considered the prevalence of anemia among Indigenous children. One is from Brazil^10^ and another includes only four countries from the region (Brazil, Guatemala, Mexico, and Venezuela)^11^. Other studies included either Indigenous children without reporting values of prevalence of anemia^12,13^ or without differentiating among Indigenous and non-Indigenous children^14–20^.

Since anemia is an indicator of both poor health and poor nutrition^2^, combining evidence from the literature about the prevalence of anemia in Indigenous children in Latin American countries can provide valuable data to governments and public health policies. Consequently, our objective was to describe the prevalence pattern of anemia among Indigenous children in Latin America.

## METHODS

### Literature Search

PRISMA guidelines were followed, and the Systematic Review was registered in PROSPERO under the number CRD42022300601. Records were identified from the databases PubMed, Google Scholar, and Lilacs by two independent researchers between May and June 2021. The search strategy combined four main categories which correspond to the inclusion criteria (a) Indigenous people, b) children, c) anemia, and d) Latin America with the Boolean operator AND. Within the main categories we used MeSH terms (if available) and free text for different variations of the category topic, for instance, “children” OR “childhood”, etc. The names of Latin American countries and the names of some representative Indigenous communities in the region were additionally included. Despite the controversies about what Latin America is and that such a position cannot be entirely correct^21^, in this study Latin America includes the following countries: Argentina, Belize, Bolivia, Brazil, Colombia, Chile, Costa Rica, Ecuador, El Salvador, French Guyana, Guyana, Guatemala, Honduras, Mexico, Nicaragua, Panamá, Paraguay, Peru, Suriname, Uruguay, and Venezuela. The spelling of countries and the Indigenous communities were varied according to differences in common use, for example “Peru” and “Perú”. However, the different spelling of the words for other places such as Latin America, Central America, and South America was not included since the searches obtained with those search terms were equal; for example, search results with the terms “Latin America” and “América Latina” had the same results. The searches were run by combining the search terms with the Boolean operator “AND”. The different terms within each of the four categories already mentioned were listed separated by the Boolean operator “OR”.

*Search terms in PubMed and Google Scholar:* (Children OR Child OR Childhood OR Infant) AND (Anemia) AND (Central America OR South America OR Latin America OR Argentina OR Belize OR Belice OR Bélice OR Bolivia OR Brazil OR Brasil OR Colombia OR Chile OR Costa Rica OR Ecuador OR El Salvador OR Guatemala OR Guyana OR Guiana OR Honduras OR Mexico OR Méjico OR México OR Mejico OR Nicaragua OR Panama OR Panamá OR Paraguay OR Peru OR Perú OR Surinam OR Uruguay OR Venezuela) AND (Indigenous OR Mapuche OR Atacameña OR Guaraní OR Guarani OR Yanomami OR Tikuna OR Ticuna OR Zenú OR Zenu OR Nasa OR Pastos OR Pasto OR Emberá OR Emberá-chamí OR Embera OR Emberachami OR Aimara OR Aymara OR Bribris OR Cabecares OR Cabecar OR Ngobes OR Ngobe OR Guaimies OR Guaimi OR Quichua OR Quechua OR Kechua OR Kichwa OR Lencas OR Lenca OR Maya OR Mayas OR Pipiles OR Pipil OR Garifuna OR Xinka OR Jinca OR Ladino OR Lempira OR Intibuca OR La Paz OR Nahuas OR Zapotecas OR Zapoteca OR Mixtecas OR Mixteca OR Otomíes OR Otomí OR Otomi OR Tzotziles OR Tzotzil OR Tzeltales OR Tzeltal OR Mazahuas OR Mazahua OR Mazatecos OR Mazateca OR Huastecos OR Huasteca OR Choles OR Chol OR Purépechas OR Purecha OR Chinantecas OR Chinanteca OR Mixes OR Tlapanecos OR Tlapaneca OR Tarahumaras OR Taraumara OR Chorotega OR Cacaopera OR Matagalpa OR Kunas OR Kuna OR Ngobe-Bugle OR Wounaan OR Naso OR Charrúa OR Wayuu OR Wayu OR Wayú).

*Search terms in Lilacs:* 214 searches were made combining the search terms, as follows: 1. “Anemia” AND “child” AND each of the countries in Latin America; 2. “Anemia” AND “children” AND each of the countries in Latin America; 3. “Anemia” AND “child” AND each of the Indigenous communities; 4. “Anemia” AND “children” AND each of the Indigenous communities; and 5. “Anemia” AND “crianças” AND “indios”.

### Inclusion and Exclusion Criteria

Studies were included if the following criteria were met: a) studied Indigenous people b) was about children (from 0 to 12 years old); c) reported a prevalence estimate of anemia; d) had been conducted in any of the countries of Latin America; e) was published either in English, Portuguese, or Spanish; f) is a peer-reviewed article; and g) was published at any date. PubMed, Google Scholar, and Lilacs searches were carried out with the defined search terms. Studies that did not met the previous criteria were not included in this systematic review. We sorted the search results in Google scholar by relevance and screened the first 200 hits, followed by the next 200 and so forth, until we were unable to find any more relevant results. Title and abstract screening were performed, and, in some cases, the methods section of the article was screened to make sure that the inclusion criteria were met. Duplicates were excluded and a full list of relevant articles was created for data extraction.

### Data Extraction and Quality Assessment and Analysis

Relevant information from selected studies was recorded in a data extraction sheet according to the following categories: Study (title, author, year, journal), geographical location (country, region), type of study, study objective, dataset, sampling technique (random or convenience), date of data collection, sample size, age group, exposure, outcome, statistical methods used, study results, study conclusions, study limitations, and study recommendations. Afterwards, the description of selected studies was assessed according to the STROBE statement^22^. Each item of the list was assigned one point: cross-sectional studies were evaluated over a total of 33 points and cohort studies over 35 points. For both types of studies, a threshold of 12 points was established to determine that they were of sufficient quality for consideration in this systematic review. The 12 points included: 1) gives a scientific background and rationale for the reported investigation; 2) states specific objectives, including any prespecified hypotheses; 3) describes the setting, locations, and relevant dates; 4) defines outcomes; 5) describes assessment methods; 6) shows the study size; 7) describes statistical methods; 8) gives the characteristics of study participants; 9) reports summary measures; 10) summarizes key results referring to study objectives; 11) gives a cautious overall interpretation of results; and 12) discuss the external validity of the study results. Thereafter, the systematic search and the study characteristics were analyzed. The descriptive analysis of the prevalence of anemia was grouped by country, by age range, and by Indigenous community.

## RESULTS

### Literature Search

**Figure 1:**
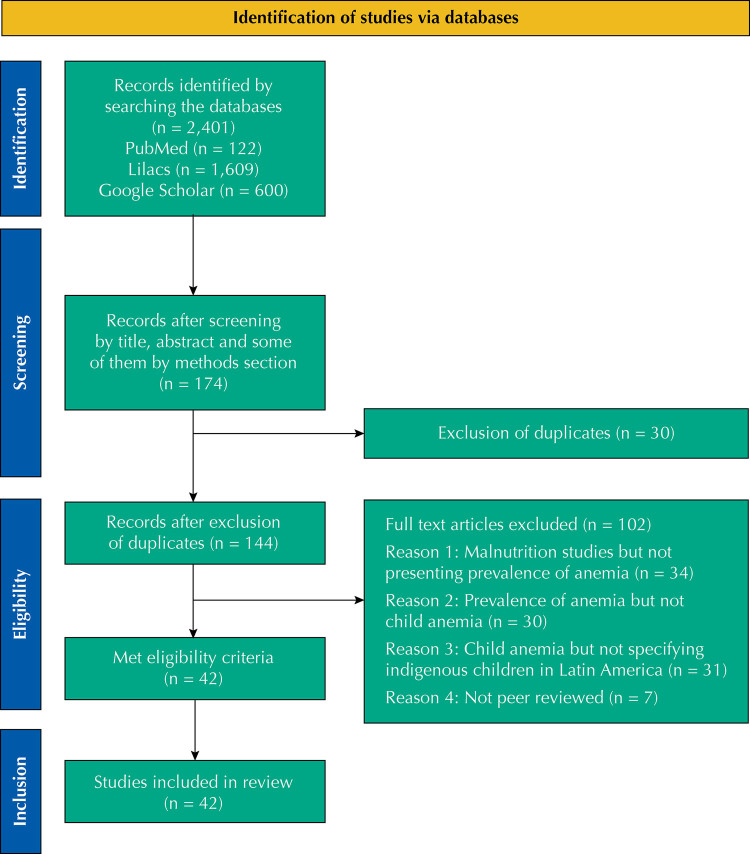
Selection process of studies included in this systematic review.

The initial search yielded 174 records (Figure 1). Out of these, 42 articles met the eligibility criteria^5,23–63^ and were included in the systematic review. A second reviewer independently replicated the searches. Scores ranged from 13 to 21 points for cross-sectional studies and the two cohort studies received scores of 21 and 35. All articles scored above the established threshold of 12 and thus were judged of good quality for inclusion in the subsequent analyses. Most articles included more than one sample taken to measure the prevalence of anemia. Therefore, Table 1 shows the different samples taken in all the studies, numbered from 1 to 133.

**Table 1:** Prevalence of anemia by Indigenous community showing the different samples taken in each study numbered from 1 to 133*.

#	Indigenous community	Country	Region	Diagnostic criteria (Hb mg/dl)^a^	Sample size	Age^a^	Total (%)^d^		95%CI	Author
							M^b^	F^c^		
1	Achuar, Urarina, Quichua	Peru	Peruvian Amazon	< 11.5	Not specified	5–11 y	45.6		-	Anticona and San Sebastian^58^ (2014)
2	Achuar, Urarina, Quichua	Peru	Peruvian Amazon	< 13.0	Not specified	12–17 y	50.9		-	Anticona and San Sebastian^58^ (2014)
3	Achuar, Urarina, Quichua	Peru	Peruvian Amazon	0–4 y: < 11.0; 5–11 y: < 11.5; ≥ 12 y: < 13.0	330	0–17 y	51.0		45.5–56.3*	Anticona and San Sebastian^58^ (2014)
4	Achuar, Urarina, Quichua	Peru	Peruvian Amazon	< 11.0	Not specified	0–4 y	56.9		-	Anticona and San Sebastian^58^ (2014)
5	Aguaruna	Peru	Districts Aramango/Imaza, Bagua province; and Cenepa, Nieva/Santiago, Condorcanqui province	< 11.0	Not specified	< 3 y	76.5		-	Huamán-Espino and Valladares^60^ (2006)
6	Aruak	Brazil	Alto Xingu, Mato Grosso state	< 11.5	78	60–120 m	61.5		50.4–71.6*	Mondini et al.^32^ (2009)
7	Aruak	Brazil	Alto Xingu, Mato Grosso state	< 11.0	71	24–60 m	62.0		50.3–72.4*	Mondini et al.^32^ (2009)
8	Aruak	Brazil	Alto Xingu, Mato Grosso state	6–59 m: < 11.0; 60–120 m: < 11.5	201	6–120 m	66.0		59.4–72.4*	Mondini et al.^32^ (2009)
9	Aruak	Brazil	Alto Xingu, Mato Grosso state	< 11.0	52	< 24 m	83.3		70.0–90.8*	Mondini et al.^32^ (2009)
10	Awajún	Peru	Provinces of Bagua and Condorcanqui, Amazon region	< 11.0	638	< 5 y	51.3		47.4–55.1	Díaz et al.^56^ (2015)
11	Aymara	Bolivia	Caranavi (Yungas)	< 14.5	12	11–12 y	0.0		0.0–24.2*	Terán et al.^24^ (2018)
12	Aymara	Bolivia	Caranavi (Yungas)	< 14.5	93	6–12 y	8.0		3.5–15.0*	Terán et al.^24^ (2018)
13	Aymara	Bolivia	Caranavi (Yungas)	< 14.5	53	8–10 y	8.0		2.5–18.4*	Terán et al.^24^ (2018)
14	Aymara	Bolivia	Caranavi (Yungas)	< 14.5	28	6–7 y	11.0		0.5–7.5*	Terán et al.^24^ (2018)
15	Aymara	Bolivia	National level	< 11.0	2,692	6–59 m	61.1		54.9–66.9	Cordero et al.^23^ (2019)
16	Aymara	Bolivia	Taraco (Puna)	< 14.5	59	8–10 y	69.0		56.8–79.8*	Terán et al.^24^ (2018)
17	Aymara	Bolivia	National level	< 11.0	2,337	6–59 m	70.2		63.6–76.1	Cordero et al.^23^ (2019)
18	Aymara	Bolivia	National level	< 11.0	1,513	6–59 m	73.8		63.1–82.2	Cordero et al.^23^ (2019)
19	Aymara	Bolivia	Taraco (Puna)	< 14.5	119	6–12 y	74.0		65.4–81.0*	Terán et al.^24^ (2018)
20	Aymara	Bolivia	Taraco (Puna)	< 14.5	35	11–12 y	74.0		57.9–85.8*	Terán et al.^24^ (2018)
21	Aymara	Bolivia	Taraco (Puna)	< 14.5	25	6–7 y	84.0		65.3–93.6*	Terán et al.^24^ (2018)
22	Aymara	Bolivia	Taraco (Puna)	< 14.5	59	11–12 y	78.0		65.7–86.8*	Terán et al.^24^ (2018)
23	Aymara	Bolivia	Taraco (Puna)	< 14.5	60	11–12 y		70.0	57.4–80.2*	Terán et al.^24^ (2018)
24	Aymara	Bolivia	Caranavi (Yungas)	< 14.5	42	6–12 y	7.0		1.8–19.7*	Terán et al.^24^ (2018)
25	Aymara	Bolivia	Caranavi (Yungas)	< 14.5	51	6–12 y		8.0	2.6–19.0*	Terán et al.^24^ (2018)
26	Brazilian Indigenous community	Brazil	North, Northeast, Central-West, Southeast/South)	< 11.0	1,117	48–59 m	32.9		30.1–35.6*	Leite et al.^5^ (2013)
27	Brazilian Indigenous community	Brazil	North, Northeast, Central-West, Southeast/South)	< 11.0	1,239	36–47 m	39.4		36.7–42.1*	Leite et al.^5^ (2013)
28	Brazilian Indigenous community	Brazil	North, Northeast, Central-West, Southeast/South)	< 11.0	1,170	24–35 m	48.8		45.9–51.7*	Leite et al.^5^ (2013)
29	Brazilian Indigenous community	Brazil	National level	< 11.0	5,397	< 60 m	51.2		47.9–54.6	Coimbra et al.^29^ (2013)
30	Brazilian Indigenous community	Brazil	North, Northeast, Central-West, Southeast/South)	< 11.0	5,397	6–59 m	51.2		49.9–52.5*	Leite et al.^5^ (2013)
31	Brazilian Indigenous community	Brazil	North, Northeast, Central-West, Southeast/South)	< 11.0	1,198	12–23 m	68.2		65.5–70.8*	Leite et al.^5^ (2013)
32	Brazilian Indigenous community	Brazil	North, Northeast, Central-West, Southeast/South)	< 11.0	673	6–11 m	80.2		77.1–83.1*	Leite et al.^5^ (2013)
33	Brazilian Indigenous community	Brazil	North, Northeast, Central-West, Southeast/South)	< 11.0	2,635	6–59 m		49.6	47.7–51.5*	Leite et al.^5^ (2013)
34	Brazilian Indigenous community	Brazil	North, Northeast, Central-West, Southeast/South)	< 11.0	2,761	6–59 m	52.8		50.9–54.7*	Leite et al.^5^ (2013)
35	Cabécar	Costa Rica	Indian reservation of Ujarrás, Puntarenas province	female: < 12.0; male: < 13.0	84	10–16 y	57.0		46.5–67.2*	Monge-Rojas et al.^44^ (2005)
36	Colombian Indigenous community	Colombia	National level	< 11.0	2,606	6–59 m	31.9		26.1–37.7	Cediel et al.^40^ (2020)
37	Ecuadorian Indigenous community	Ecuador	Riobamba canton of Chimborazo province	< 11.5	36	5–6 y	18.8		9.8–35.0*	Robalino et al.^47^ (2017)
38	Ecuadorian Indigenous community	Ecuador	National level	< 11.0	1,236	6–59 m	37.8		27.9–48.9	Ramírez-Luzurriaga et al.^46^ (2020)
39	Embera-Chamí	Colombia	Caldas	5–11 y: < 11.5	82	6–10 y	8.6		3.9–16.8*	Cardona et al.^43^ (2014)
40	Embera-Chamí	Colombia	Caldas	6–59 m: < 11.0; 5–11 y: < 11.5	80	0–5 y	11.0		5.8–20.2*	Cardona et al.^43^ (2014)
41	Embera-Chamí	Colombia	Caldas	5–11 y: < 11.5; 12–14 y: < 12.0	22	10–15 y	36.4		19.7–57.0*	Cardona et al.^43^ (2014)
42	Embera-Chamí	Colombia	Caldas	5–11 y: < 11.5; 12–14 y: < 12.0	94 boys / 90 girls	0–15 y	12.0	14.3	Boys: 6.5–19.9 Girls: 8.5–23.3*	Cardona et al.^43^ (2014)
43	Guaraní	Brazil	Rio de Janeiro (Sapukai, Parati-Mirim, Araponga, Sítio Rio Pequeno and Mamanguá) and São Paulo (Boa Vista)	< 11.0	128	6–11 m	88.9		82.4–93.5*	Barreto et al.^28^ (2014)
44	Guatemalan Indigenous community	Guatemala	National level	< 11.0	3,861	6–59 m	49.6		46.4–52.8*	Ramírez-Zea et al.^48^ (2014)
45	Kamaiurá	Brazil	Alto Xingu, Mato Grosso state	< 11.0	38	24–60 m	50.0		34.8–65.2*	Mondini et al.^34^ (2007)
46	Kamaiurá	Brazil	Alto Xingu, Mato Grosso state	< 11.5	50	60–120 m	50.0		36.6–63.4*	Mondini et al.^34^ (2007)
47	Kamaiurá	Brazil	Alto Xingu, Mato Grosso state	6–59 m: < 11.0; 5–10 y: < 11.5	104	< 10 y	55.3		46.2–64.9*	Mondini et al.^34^ (2007)
48	Kamaiurá	Brazil	Alto Xingu, Mato Grosso state	< 11.0	16	6–24 m	81.3		57.0–93.4*	Mondini et al.^34^ (2007)
49	Kamaiurá	Brazil	Alto Xingu, Mato Grosso state	6–59 m: < 11.0; 5–10 y: < 11.5	45	< 10 y	64.4		49.8–76.8*	Mondini et al.^34^ (2007)
50	Kamaiurá	Brazil	Alto Xingu, Mato Grosso state	6–59 m: < 11.0; 5–10 y: < 11.5	59	< 10 y		47.5	35.3–60.0*	Mondini et al.^34^ (2007)
51	Karapotó	Brazil	Aldeia Plak-Ô and povoado Terra Nova, in the municipality of São Sebastião, Alagoas	< 11.0	61	25–59 m	49.1		37.1–61.4*	Pereira et al.^30^ (2012)
52	Karapotó	Brazil	Aldeia Plak-Ô and povoado Terra Nova, in the municipality of São Sebastião, Alagoas	< 11.0	99	6–59 m	57.6		47.7–66.9*	Pereira et al.^30^ (2012)
53	Karapotó	Brazil	Plak-Ô reservation and povoado Terra Nova, in the municipality of São Sebastião, Alagoas	< 11.0	97	6–59 m	57.7		47.8–67.1*	Campos et al.^27^ (2016)
54	Karapotó	Brazil	Aldeia Plak-Ô and povoado Terra Nova, in the municipality of São Sebastião, Alagoas	< 11.0	33	6–24 m	72.7		55.8–84.9*	Pereira et al.^30^ (2012)
55	Karapotó	Brazil	Aldeia Plak-Ô and povoado Terra Nova, in the municipality of São Sebastião, Alagoas	< 11.0	46	6–59 m		56.5	42.2–69.8*	Pereira et al.^30^ (2012)
56	Karapotó	Brazil	Aldeia Plak-Ô and povoado Terra Nova, in the municipality of São Sebastião, Alagoas	< 11.0	53	6–59 m	58.4		45.1–70.8*	Pereira et al.^30^ (2012)
57	Karibe	Brazil	Alto Xingu, Mato Grosso state	< 11.0	85	24–60 m	66.2		55.3–75.1*	Mondini et al.^32^ (2009)
58	Karibe	Brazil	Alto Xingu, Mato Grosso state	< 11.5	116	60–120 m	67.3		58.3–75.1*	Mondini et al.^32^ (2009)
59	Karibe	Brazil	Alto Xingu, Mato Grosso state	6–59 m: < 11.0; 60–120 m: < 11.5	269	6–120 m	70.1		64.5–75.4*	Mondini et al.^32^ (2009)
60	Karibe	Brazil	Alto Xingu, Mato Grosso state	< 11.0	68	6–24 m	85.0		74.8–92.0*	Mondini et al.^32^ (2009)
61	Kaxinanuá	Brazil	Município de Jordão, in the valley of Juruá	< 11.0	56	6–59 m	37.5		26.0–50.6*	Oliveira et al.^31^ (2011)
62	Ma- cro-Jê	Brazil	Paraná (Kreen-Aka-rôre)-village Nacipotire	< 11.0	36	6–59 m	33.3		20.2–49.7*	Baruzzi et al.^36^ (2001)
63	Ma- cro-Jê	Brazil	Paraná (Kreen-Aka-rôre)-village Nacipotire	< 11.0	82	6 m – 14 y	56.1		45.3–66.3*	Baruzzi et al.^36^ (2001)
64	Ma- cro-Jê	Brazil	Paraná (Kreen-Aka-rôre)-village Nacipotire	< 12.0	46	6–14 y	73.9		59.6–84.5*	Baruzzi et al.^36^ (2001)
65	Mapuche	Chile	Cautín province	< 11.0	110	8–14 m	4.5		36.5–54.8*	Franco et al.^37^ (1987)
66	Mapuche	Chile	Cautín province	< 11.0	140	8–15 m	4.5		1.8–9.2*	Franco et al.^38^ (1990)
67	Mapuche	Chile	Cautín province	< 11.0	76	12 m	29		19.9–40.0*	Franco et al.^39^ (1996)
68	Mexican Indigenous community	Mexico	National level	< 11.5	918	5–11 y	10.4		8.2–13.2	De la Cruz-Góngora et al.^51^ (2013)
69	Mexican Indigenous community	Mexico	National level	< 11.5	647	5–11 y	11.8		8.9–15.4	De la Cruz-Góngora et al.^51^ (2013)
70	Mexican Indigenous community	Mexico	National level	< 9.5	27	6–11 m	16.2		9.2–23.2	Villapando et al.^55^ (2003)
71	Mexican Indigenous community	Mexico	National level	< 11.5	1,067	5–11 y	17.9		15.6–20.6	De la Cruz-Góngora et al.^51^ (2013)
72	Mexican Indigenous community	Mexico	National level	< 12.0	127	11 y	17.9		13.5–22.2	Villapando et al.^55^ (2003)
73	Mexican Indigenous community	Mexico	National level	< 12.0	294	9–10 y	22.6		19.1–26.1	Villapando et al.^55^ (2003)
74	Mexican Indigenous community	Mexico	National level	< 11.0	155	48–59 m	23.0		15.4–30.6	Villapando et al.^55^ (2003)
75	Mexican Indigenous community	Mexico	National level	12–71 m: < 11.0; 6–11 y: < 12.0	1,068	5–11 y	24.0		21.3–26.7	Villapando et al.^55^ (2003)
76	Mexican Indigenous community	Mexico	National level	< 11.0	1,032	6–59 m	24.8		21.1–28.9	Batis et al.^49^ (2020)
77	Mexican Indigenous community	Mexico	National level	< 11.0	356	12–59 m	25.8		20.0–32.6	De la Cruz-Góngora et al.^51^ (2013)
78	Mexican Indigenous community	Mexico	National level	< 12.0	312	7–8 y	25.8		21.6–30.0	Villapando et al.^55^ (2003)
79	Mexican Indigenous community	Mexico	National level	< 11.0	909	12–59 m	25.9		22.2–30.0	De la Cruz-Góngora et al.^51^ (2013)
80	Mexican Indigenous community	Mexico	National level	12–71 m: < 11.0; 6–11 y: < 12.0	335	5–6 y	26.1		21.4–30.4	Villapando et al.^55^ (2003)
81	Mexican Indigenous community	Mexico	National level	< 11.0	902	12–59 m	27.0		22.8–31.7	Palacios-Rodríguez et al.^50^ (2019)
82	Mexican Indigenous community	Mexico	National level	< 11.0	163	36–47 m	30.5		24.6–36.5	Villapando et al.^55^ (2003)
83	Mexican Indigenous community	Mexico	Rural	< 11.0	385	6–59 m	31.6		27.2–36.5*	Rivera et al.^54^ (2003)
84	Mexican Indigenous community	Mexico	South	< 11.0	429	6–59 m	34.3		29.9–38.9*	Rivera et al.^54^ (2003)
85	Mexican Indigenous community	Mexico	National level	< 11.0	605	6–59 m	35.2		31.5–39.1*	Rivera et al.^54^ (2003)
86	Mexican Indigenous community	Mexico	National level	6–11 m: < 9.5; 12–71 m < 11.0	614	6–59 m	35.8		32.0–39.5	Villapando et al.^55^ (2003)
87	Mexican Indigenous community	Mexico	Other regions	< 11.0	176	6–59 m	37.5		30.7–44.9*	Rivera et al.^54^ (2003)
88	Mexican Indigenous community	Mexico	National level	< 11.0	587	12–59 m	40.2		35.7–44.8	De la Cruz-Góngora et al.^51^ (2013)
89	Mexican Indigenous community	Mexico	Urban	< 11.0	220	6–59 m	40.3		34.2–47.1*	Rivera et al.^54^ (2003)
90	Mexican Indigenous community	Mexico	National level	< 11.0	149	24–35 m	42.0		35.5–48.5	Villapando et al.^55^ (2003)
91	Mexican Indigenous community	Mexico	Mountain: Mexico; highlands: Veracruz; coast: Hidalgo	< 11.0	194	6–24 m	52.6		39.7–69.3	Aguirre-Arenas et al.^52^ (2013)
92	Mexican Indigenous community	Mexico	National level	< 11.0	120	12–23 m	57.0		50.6–65.2	Villapando et al.^55^ (2003)
93	Nasa (Resguardo San Lorenzo, de Caldono)	Colombia	Cauca department	< 11.0	Not specified	1–3 y	16.1		-	Gaviria et al.^41^ (2017)
94	Nasa (Resguardo San Lorenzo, de Caldono)	Colombia	Cauca department	< 11.0	62	1–5 y	21.0		12.5–32.8*	Gaviria et al.^41^ (2017)
95	Nasa (Resguardo San Lorenzo, de Caldono)	Colombia	Cauca department	< 11.0	Not specified	4–5 y	25.8		-	Gaviria et al.^41^ (2017)
96	Nasa, Yanacona, Quichua, Inga, Kofan and Misak (Guambiano)	Colombia	Cali	< 11.0	551	5–14 y	25.8		22.3–29.6*	Bolaños et al.^42^ (2014)
97	Peruvian Indigenous community	Peru	National level	< 11.0	172	6–59 m	43.5		32.0–59.0	Flórez-Bendezú et al.^57^ (2015)
98	Piaroa	Venezuela	Atures Municipality, 50 km north of Puerto Ayacucho	< 11.0	9	1–3 y		100.0	70.1–100*	García-Casal et al.^62^ (2008)
99	Piaroa	Venezuela	Atures Municipality, 50 km north of Puerto Ayacucho	< 11.0	10	1–3 y	100.0		72.2–100*	García-Casal et al.^62^ (2008)
100	Piaroa	Venezuela	Atures Municipality, 50 km north of Puerto Ayacucho	< 11.5	31	4–10 y		100.0	89.0–100*	García-Casal et al.^62^ (2008)
101	Piaroa	Venezuela	Atures Municipality, 50 km north of Puerto Ayacucho	< 11.5	23	4–10 y	91.0		73.2–97.6*	García-Casal et al.^62^ (2008)
102	Quechua	Bolivia	National level	< 11.0	1,513	6–59 m	55.0		49.3−60.5	Cordero et al.^23^ (2019)
103	Quechua	Bolivia	National level	< 11.0	2,692	6–59 m	56.7		51.8–61.6	Cordero et al.^23^ (2019)
104	Quechua	Bolivia	National level	< 11.0	2,337	6–59 m	66.4		61.4−71.1	Cordero et al.^23^ (2019)
105	Shuar	Ecuador	Morona-Santiago province: Upano Valley, Cross-Cutucú	< 11.5	653	5–11 y	10.9		8.7–13.5*	De Louize et al.^45^ (2021)
106	Shuar	Ecuador	Morona-Santiago province: Upano Valley, Cross-Cutucú	< 12.0	164	12–14 y	11.0		7.0–16.8*	De Louize et al.^45^ (2021)
107	Shuar	Ecuador	Morona-Santiago province: Upano Valley, Cross-Cutucú	< 11.0	127	0–4 y	20.4		14.3–28.4*	De Louize et al.^45^ (2021)
108	Suruí	Brazil	Sete de Setembro Indigenous Land, in Rondônia, Mato Grosso	6–59 m: < 11.0; 60–119 m: < 11.5	268	6–119 m	80.6		75.4–84.9*	Orellana et al.^33^ (2006)
109	Taraumara	Mexico	Northern Mexico (municipios de Guachochi, Balleza and Batopilas)	< 11.5	154	9–11 y	10.4		6.4–16.3*	Monarrez-Espino et al.^53^ (2004)
110	Taraumara	Mexico	Northern Mexico (municipios de Guachochi, Balleza and Batopilas)	6–11 y: < 11.5; 12–13 y + female 14 y: < 12.0; boys 14 y: < 13.0	331	6–14 y	13.0		9.8–17.1*	Monarrez-Espino et al.^53^ (2004)
111	Taraumara	Mexico	Northern Mexico (municipios de Guachochi, Balleza and Batopilas)	< 11.5	125	6–8 y	14.4		9.2–21.7*	Monarrez-Espino et al.^53^ (2004)
112	Taraumara	Mexico	Northern Mexico (municipios de Guachochi, Balleza y Batopilas)	12–13 y + female 14 y: < 12.0; boys 14 y: < 13.0	52	12–14 y	17.3		9.2–30.0*	Monarrez-Espino et al.^53^ (2004)
113	Terena	Brazil	Terenas villages of Limão Verde, Córrego Seco, district of Aquidauana, Mato Grosso do Sul state	6–72 m: < 11.0; 73–120 m: < 11.5	59	60–120 m	40.7		29.1–53.4*	Morais et al.^35^ (2005)
114	Terena	Brazil	Terenas villages of Limão Verde and Córrego Seco, district of Aquidauana, Mato Grosso do Sul state	< 11.0	65	24–60 m	50.8		38.9–62.5*	Morais et al.^35^ (2005)
115	Terena	Brazil	Terenas villages of Limão Verde and Córrego Seco, district of Aquidauana, Mato Grosso do Sul state	6–72 m: < 11.0; 73–120 m: < 11.5	167	6–120 m	62.3		54.7–69.3*	Morais et al.^35^ (2005)
116	Terena	Brazil	Terenas villages of Limão Verde and Córrego Seco, district of Aquidauana, Mato Grosso do Sul state	< 11.0	43	6–24 m	86.1		72.4–93.8*	Morais et al.^35^ (2005)
117	Xavante	Brazil	Villages, Pimentel Barbosa and Etênhiritipá, Mato Grosso state, Central Brazil	6–59 m: 10.0–10.9 g/dL; 5–11 y: 11.0–11.4 g/dL	Not specified	6–59 m	23.6		-	Welch et al.^25^ (2020)
118	Xavante	Brazil	Villages, Pimentel Barbosa and Etênhiritipá, Mato Grosso state, Central Brazil	5–11 y: 11.0–11.4 g/dL; 12–14 y: 11.0–11.9 g/dL; females nonpregnant ≥ 15 y: 11.0–11.9 g/dL; male ≥ 15 y: 11.0–12.9g/dL	Not specified	5–9 y	23.3		-	Welch et al.^25^ (2020)
119	Xavante	Brazil	Pimentel Barbosa Indigenous Reserve	< 11.0	89	2 to < 5 y	50.5		40.4–60.7*	Ferreira et al.^26^ (2017)
120	Xavante	Brazil	Pimentel Barbosa Indigenous Reserve	6–59 m: < 11.0; 5–11 y: < 11.5	257	> 6 m to < 10 y	50.8		44.9–50.7*	Ferreira et al.^26^ (2017)
121	Xavante	Brazil	Pimentel Barbosa Indigenous Reserve	< 11.0	143	< 5 y	62.2		54.1–69.8*	Ferreira et al.^26^ (2017)
122	Xavante	Brazil	Pimentel Barbosa Indigenous Reserve	< 11.0	54	> 6 m to < 2 y	77.8		64.9–86.9*	Ferreira et al.^26^ (2017)
123	Xavante	Brazil	Pimentel Barbosa Indigenous Reserve	< 11.5	115	5–10 y	20.0	18.3	-	Ferreira et al.^26^ (2017)
124	Yanomami and Warao	Venezuela	Orinoco Delta	< 8 y: < 11.5; 8–11 y: < 11.9; males 12–14 y: < 12.5; females 12–14 y: < 11.8; males > 14 y: < 13.3; females > 14 y: < 12	152	4–16 y	9.0		5.5–15.0*	Verhagen et al.^61^ (2013)
125	Yanomami and Warao	Venezuela	Amazon Region	< 8 y: < 11.5; 8–11 y: < 11.9; males 12–14 y: < 12.5; females 12–14 y: < 11.8; males > 14 y: < 13.3; females > 14 y: <12	133	4–16 y	24.0		17.6–32.0*	Verhagen et al.^61^ (2013)
126	Yomybato	Peru	Madre de Dios	5–11 y: < 11.5; 12–14 y: < 12.0; > 15 y: < 13.0	117	5–19 y	26.6		17.8–39.2	Cabada et al.^59^ (2014)
127	Yomybato	Peru	Madre de Dios	< 11.0	62	6–59 m	67.3		51.9–85.0	Cabada et al.^59^ (2014)
128	Yucpa	Venezuela	Zulia state (Aroy)	1–2 y: < 10.7; 3–5 y: < 10.9	Not specified	< 6 y	25.0		-	Diez et al.^63^ (1999)
129	Yucpa	Venezuela	Zulia state (Marewa)	1–2 y: < 10.7; 3–5 y: < 10.9	Not specified	< 6 y	38.9		-	Diez et al.^63^ (1999)
130	Yucpa	Venezuela	Zulia state (Marewa)	< 11.5	Not specified	6–11 y	56.4		-	Diez et al.^63^ (1999)
131	Yucpa	Venezuela	Zulia state (Peraya)	1–2y: < 10.7; 3–5 y: < 10.9	Not specified	< 6 y	60.0		-	Diez et al.^63^ (1999)
132	Yucpa	Venezuela	Zulia state (Aroy)	< 11.5	Not specified	6–11 y	66.7		-	Diez et al.^63^ (1999)
133	Yucpa	Venezuela	Zulia state (Peraya)	< 11.5	Not specified	6–11 y	72.0		-	Diez et al.^63^ (1999)

a y: age in years, m: age in months.

b m = males.

c f = females.

d Values in the middle are overall values.

* Confidence intervals that were lacking in the articles were calculated by the authors of this systematic review based on the available point estimate and sample size and are marked on the table with an asterisk (*). This was done at https://epitools.ausvet.com.au/ciproportion and the calculation methods were Wilson for samples of size n < 40 and the Agresti-Coull method for samples of size n ≥ 40.

### General Characteristics of the Studies

Selected articles were written one the following three languages: English (26, 61.9%), Spanish only (11, 26.2%), Portuguese only (5, 11.9%). The oldest article was published in 1987 and the newest in 2021. The articles covered 10 countries (Bolivia, Brazil, Chile, Colombia, Costa Rica, Ecuador, Guatemala, Mexico, Peru, and Venezuela), and the studies were conducted in 30 specific regions in Latin America. Cross-sectional studies were 40 (95.2%) and cohort studies, two^25,39^. Ten studies^23,40,46,48–51,54,55,57^ used data from national databases such as the Mexican National Health and Nutrition Survey from 2012 or the 2010 Colombian Demographic and Health Survey.

The sample sizes diverged widely across the studies. The smallest sample size was 36^47^, whereas the largest sample sizes were from studies including secondary data from national databases; for example, the highest number was from the National Maternal and Child Health Surveys in Guatemala (n = 5,735)^48^. Age ranges varied greatly: some studies considered children only within a narrow age group, e.g., 6–24 months^52^, whereas other studies, included broader age ranges, e.g., 0–17 years old^58^. Most of the studies lacked separated data for boys and girls.

De Louize et al. carried out the most recent data collection on the prevalence of anemia in indigenous communities in 2017^45^, followed by two articles that collected data in 2013^23,57^; all other studies collected data before 2013. Thirteen studies did not specify the date of data collection. Not all studies mentioned the name of the investigated Indigenous community. For example, studies including data from national databases considered the whole Indigenous population of the country without specifying the names of the different communities. The 42 articles of this systematic review included 39 Indigenous communities from Latin America (Table 2).

**Table 2:** Distribution of articles by Indigenous community.

#	Indigenous community	Country	Region	Author
1	Achuar	Peru	Peruvian Amazon	Anticona and San Sebastian^58^ (2014)
2	Aguaruna	Peru	Districts of Aramango and Imaza in the province of Bagua; and Cenepa, Nieva and Santiago in the province of Condorcanqui	Huamán-Espino and Valladares^60^ (2006)
3	Aruak	Brazil	Alto Xingu, Mato Grosso state	Mondini et al.^32^ (2009)
4	Awajún	Peru	Provinces of Bagua and Condorcanqui in the Amazon region	Díaz et al.^56^ (2015)
5	Aymara	Bolivia	El Alto, Caranavi (Yungas), Taraco (Puna)	Terán et al.^24^ (2018)
8	Cabécar	Costa Rica	Indian reservation of Ujarrás, Puntarenas	Monge-Rojas et al.^44^ (2005)
10	Embera-Chamí	Colombia	Caldas	Cardona et al.^43^ (2014)
11	Guaraní	Brazil	Rio de Janeiro (Sapukai, Parati-Mirim, Araponga, Sítio Rio Pequeno, and Mamanguá) and São Paulo (Boa Vista)	Barreto et al.^28^ (2041)
12	Inga	Colombia	Cali	Bolaños et al.^42^ (2014)
13	Kamaiurá	Brazil	Alto Xingu, Mato Grosso state	Mondini et al.^34^ (2007)
14	Karapotó	Brazil	Plak-Ô reservation and Terra Nova settlement, in São Sebastião, Alagoas state	Campos et al.^27^ (2016), Pereira et al.^30^ (2012)
16	Karibe	Brazil	Alto Xingu, Mato Grosso state	Mondini et al.^32^ (2009)
17	Kaxinanuá	Brazil	Município de Jordão, in the valley of Juruá	Oliveira et al.^31^ (2011)
18	Kofan	Colombia	Cali	Bolaños et al.^42^ (2014)
19	Ma-cro-Jê	Brazil	Paraná (Kreen-Aka-rôre)-village Nacipotire	Baruzzi et al.^36^ (2001)
20	Mapuche	Chile	Cautín province	Franco et al.^39^ (1996)
22	Misak (Guambiano)	Colombia	Cali	Bolaños et al.^42^ (2014)
23	Nasa (Resguardo San Lorenzo, de Caldono)	Colombia	Cauca department	Gaviria et al.^41^ (2017)
24	Nasa	Colombia	Cali	Bolaños et al.^42^ (2014)
25	Piaroa	Venezuela	Atures municipality, located 50 kilometers north of Puerto Ayacucho	García-Casal et al.^62^ (2008)
26	Quechua	Peru, Bolivia, Colombia	Peruvian Amazon; Cali, Colombia	Anticona and San Sebastian^58^ (2014), Bolaños et al.^42^ (2014), Cordero et al.^23^ (2019)
28	Shuar	Ecuador	Morona-Santiago province: Upano Valley and Cross-Cutucú	De Louize et al.^23^ (2021)
29	Suruí	Brazil	Sete de Setembro Indigenous Land, in Rondônia and Mato Grosso	Orellana et al.^33^ (2006)
30	Taraumara	Mexico	Northern Mexico (municipalities of Guachochi, Balleza and Batopilas)	Monarrez-Espino et al.^53^ (2004)
31	Terena	Brazil	Terenas villages of Limão Verde and Córrego Seco, district of Aquidauana, in Mato Grosso do Sul state	Morais et al.^35^ (2005)
32	Urarina	Peru	Peruvian Amazon	Anticona and San Sebastian^58^ (2014)
33	Warao	Venezuela	Amazon Region	Verhagen et al.^61^ (2013)
34	Xavante	Brazil	Pimentel Barbosa Indigenous Reserve and Etênhiritipá, Mato Grosso state, Central Brazil	Ferrerira et al.^26^ (2017), Welch et al.^25^ (2020)
36	Yanacona	Colombia	Cali	Bolaños et al.^42^ (2014)
37	Yanomami	Venezuela	Amazon Region	Verhagen et al.^61^ (2013)
38	Yomybato	Peru	Madre de Dios	Cabada et al.^59^ (2014)
39	Yucpa	Venezuela	Zulia state (Aroy)	Diez et al.^63^ (1999)

Prevalence of anemia was the outcome measured for this systematic review and all the studies presented it as a percentage. The main diagnostic tool used to identify anemia was the Hemocue^64^, and the other methods used were: Cyanmethemoglobin method^38,39,44,47^, Automated biometry^46^, Haemogram test^43^, Ultralogic 800^37^, Coulter counter^62^, EDTA tube^61^, and the Chromogenic method^63^. Moreover, almost all the studies followed the recommendations of the World Health Organization to diagnose anemia among children (for children from 6 to 59 months: < 11.0 mg/dL; for children from 5 to 11 years old: < 11.5 mg/dL; and for children from 12 to 14 years old: < 12.0 mg/dL) (1). Two studies used other diagnostic thresholds^55,63^ (Table 1).

### Prevalence of Anemia by Country and Indigenous Community

**Figure 2:**
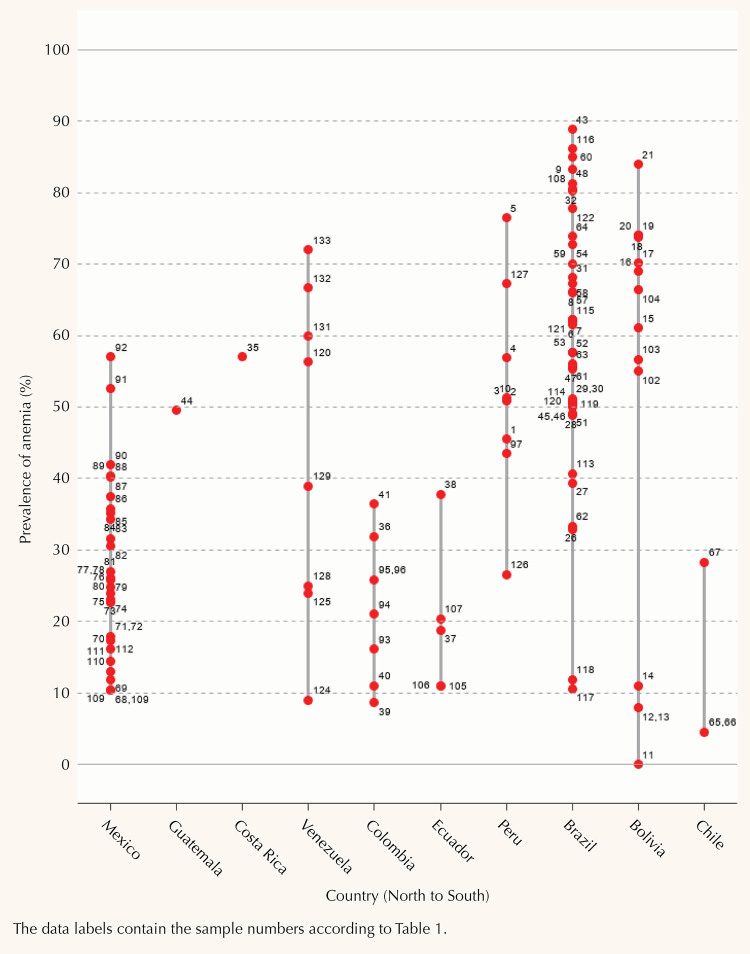
Prevalence of anemia by country.

Bolivia, Brazil, and Peru showed the highest overall prevalence of anemia (Figure 2), with the highest values among Guaraní children from 6 to 11 months (88.9%) in five villages in the state of Rio de Janeiro (Sapukai, Parati-Mirim, Araponga, Sítio Rio Pequeno, and Mamanguá) and one village in the state of São Paulo (Boa Vista) in Brazil^28^. The lowest prevalence of anemia (0%) was observed in Bolivia among children between 11 and 12 years from the Aymara Indigenous community in Caranavi in the Taraco district situated on the shores of Lake Titicaca at a high altitude^24^. The second lowest prevalence of anemia including boys and girls (4.5%) was found in Chile, among Mapuche children from 8 to 14 months old from rural areas in the province of Cautín^37^. We found that the prevalence of anemia among Indigenous children has been reported in 10 countries in Latin America. However, almost one-third of the studies were from Brazil (13 studies). Also, from the 39 Indigenous communities included in this systematic review, only four were part of more than one study: Aymara (2 studies in Bolivia)^24,65^, Quechua (1 study in Bolivia^23^, 1 study in Colombia^42^, and 1 study in Peru^58^), Karapotó (2 studies in Brazil^27,30^), and Xavante (2 studies in Brazil^25,26^).

**Figure 3:**
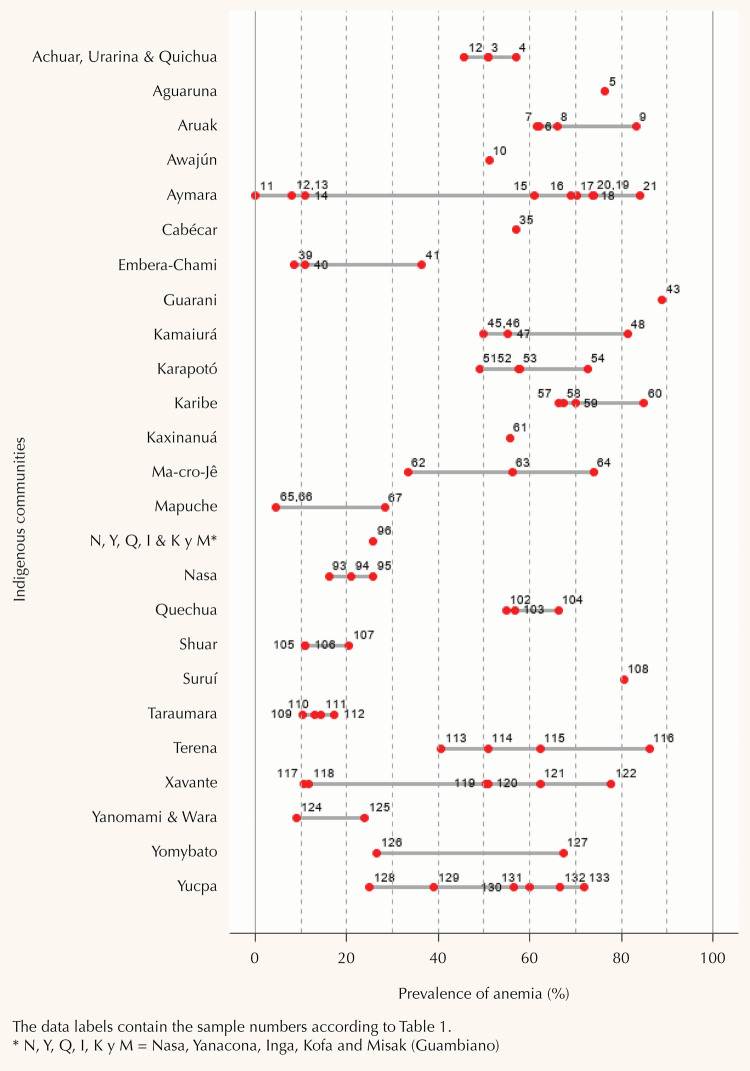
Prevalence of anemia by Indigenous community.

Prevalence of anemia among children is a severe public health problem in 54% (21 out of 39) of the Indigenous communities in Latin America included in this systematic review (prevalence ≥ 40%)^4^. The Indigenous communities that showed prevalence of anemia above 40% were the Aymara (Bolivia); Aruak, Guaraní, Kamaiurá, Karapotó, Karibe, Kaxinanuá, Ma-cro-Jê, Suruí, Terena, Xavante (Brazil); Cabécar (Costa Rica); Achuar, Aguaruna, Awajún, Urarina, Yomybato (Peru); Piaroa and Yucpa (Venezuela); and Quechua (Peru and Bolivia) (Figure 3). The Aguaruna in Peru; the Guaraní, Kamaiurá, Karibe, Suruí, and Xavante in Brazil; the Aymara in Bolivia; and the Yucpa in Venezuela are Indigenous communities registering prevalence of anemia above 70%, leading the list in terms of anemia as a severe public health problem for young children.

## DISCUSSION

This systematic review revealed an alarming situation regarding anemia in Indigenous communities in Latin America. Data obtained from Brazil confirm the gravity of the situation in the Indigenous population, with extremely high prevalence rates among children as described by Lício, Fávaro and Chaves^10^, who allege that such information is essential to contribute to health care priorities in these communities.

We found that the prevalence of anemia among Indigenous children has been reported in 10 countries in Latin America. However, almost one-third of the studies were from Brazil (13 studies). As Licio et al.^10^ stated, since 2001, studies investigating the occurrence of anemia in Indigenous populations increased significantly in Brazil, occupying an important space in the health status and social inequalities debate. Indigenous communities in Brazil correspond to 0.5% of the entire population (820,000 inhabitants), whereas, for example, 41% of the country population in both Guatemala (5,880,000) and Bolivia (4,120,000) is considered Indigenous^6^. Only one study from Guatemala and two from Bolivia could be included in this systematic review. However, we reported seven studies from Mexico and five from Peru which are the two countries with the highest numbers of Indigenous people in Latin America, namely 16,830,000 (40.3%) and 7,600,000 (18.2%) from a total Indigenous population of 41,810,000^6^. In addition, from the 39 Indigenous communities included in this systematic review, only four of them appeared in more than one study: Aymara in Bolivia; Quechua in Bolivia, Colombia, and Peru; Karapotó and Xavante in Brazil. These results suggest that the data on specific Indigenous communities is scarce. Therefore, a consistent, collaborative approach is crucial to deliver medical research and care of Indigenous communities^66^.

As stated above, numerous factors hinder the determination of the exact number of Indigenous people in Latin America; however, considering the existence of approximately 780 Indigenous groups^6^, this means that this systematic review covered 5.0% of the Indigenous communities in the region. Therefore, studies about the situation of specific Indigenous communities in the region are lacking. We found studies about the prevalence of anemia among children but without making any distinction between non-Indigenous and Indigenous children: for example, a study by Vázquez et al. in 2019^14^ showed that the overall prevalence of anemia in children from Latin America in general is 28.56%, and Mujica et al.^16^ found anemia prevalences from 4.0% to no more than 61.3% in children under six years of age based on 2014 data in Latin America and the Caribbean. Several researchers acknowledge that they have underrepresented Indigenous populations in Latin America, with significant underrepresentation of those groups in their research^17^. We also found that studies including national databases present lower values of anemia prevalence among children compared with the studies specifically targeting Indigenous communities. Using data at the national level provides reliable information only for large geographic domains, thus it can provide information at the national level for the central government, but has less reliability for regional governments and is useless for local governments. Thus, governments and private organizations in Latin American countries should be aware that national databases could be masking extreme prevalences of anemia in vulnerable populations.

Furthermore, recent studies are lacking. Data for studies in all countries, except for Brazil, was outdated. Our findings show that the most recent data collection on prevalence of anemia from Indigenous communities was done in 2017, followed by 2016 and 2013. All other studies collected data before 2013, which means that currently (2021) recent data on the prevalence of anemia among Indigenous children in Latin America is awfully scarce. Moreover, reports covering a wide range of years are lacking. Since almost all the studies included in this systematic review were cross-sectional, possible causes of the prevalence of anemia cannot be determined. On the other hand, the lack of studies with longer-term follow-ups of specific Indigenous communities hinders monitoring of the course of child anemia and comparing anemia between different countries and regions.

*Age groups:* Our results indicate that the youngest children reported higher prevalence of anemia. The highest values were observed in the age groups between 6 and 35 months. Older children also showed high prevalence of anemia, but not as high as the youngest. For example, particularly among the Aruak people in Brazil, a very high number of children between six and 23 months old show moderate or severe anemia when compared with those between 24 and 59 months^32^. In the Peruvian Amazon, anemia was more prevalent in the 0 to 5-year age group from the Achuar, Urarina, and Quichua Indigenous communities^58^.

*Risk factors:* This systematic review suggests several risk factors for high prevalence of anemia among Indigenous communities in Latin America. Babyar^66^ affirms that Indigenous populations in Latin America continue to suffer from health disparities compared with general populations. Particularly, in Brazil, Bolivia, Colombia, Ecuador, Peru, Paraguay, and Venezuela, approximately 200 Indigenous communities live in voluntary isolation, and the pressure over natural resources on their territory or in nearby areas puts them at risk of extreme vulnerability^7^. According to Díaz Arana et al.^56^ living conditions, access to healthcare, and nutritional status of children under five years of age are markedly different between the Indigenous population and the non-Indigenous population in Peru. Likewise, Ferreira et al.^26^ found that Xavante children's high rates of anemia reveal an ethnic disparity between them and the Brazilian population in general, suggesting the causes of anemia are largely dependent on complex, variable relationships between socioeconomic, sociodemographic, and biological factors. In general, Indigenous peoples in Latin America have very limited access to health care services, often lacking geographical access, which means the closest health ward is far away from their communities. Moreover, they usually face obstacles in accessing health services due to mistrust generated by a history of racism and structural discrimination^67^.

Besides, specific nutritional causes can be considered as risk factors for high prevalence of anemia. Franco et al.^37^ suggested that maternal milk might have a protective biologic role in preventing iron deficiency anemia among the Mapuche Indigenous community in Chile. Furthermore, Mujica et al.^68^ showed recently that the low prevalence of anemia in Chile is very likely a result of the iron-fortified milk provided by the National Complementary Feeding Program. The Aguarunas in Peru are also an example in which anemia prevalence is associated with their diet, based primarily on cassava and bananas, with little animal protein^60^. On the other hand, in the case of the Yucpa Indigenous community in Venezuela, the high frequency of anemia without nutritional cause is attributed to the prevalence of infectious diseases such as hepatitis, parasites, and skin and respiratory tract infections^63^.

*Prevention:* Khambalia et al.^11^ identified iron deficiency, malaria, and helminth infections as the three main causes of anemia among Indigenous populations, which can be addressed by a combination of interventions, such as fortification of staple foods with iron and other micronutrients, iron supplements targeted to risk groups, use of insecticide-treated materials and bed nets, deworming (anthelminthics) in risk groups, and prevention and treatment of malaria. Anemia can also be prevented and controlled by fully immunizing children; treating communicable diseases; managing obstetric complications, particularly excessive bleeding; and using modern family planning methods. For the first six months of a child's life, breastfeeding must be the sole basis for feeding, iron-rich foods should be given as a supplement, and sanitation facilities must be improved^11^.

*Public policies:* Several health programs and policies have been implemented in Latin American countries in the past two decades to generally address anemia as a public health problem^14^. However, Indigenous communities continue experiencing difficult situations. According to CEPAL^9^, Guatemala has the highest proportion of Indigenous population living in municipalities with high or critical vulnerability (77.9%); Colombia (65.8%) follows in the list, then Mexico (38.8%), Peru (33%), and finally Chile (20.9%). Beyond this variability, a common pattern is the inequality affecting Indigenous peoples, with the widest gaps found in Colombia and Mexico. Although inequalities are also systematic within municipalities, the vulnerability index among Indigenous populations seems to always be higher than that estimated for non-Indigenous populations^9^. The Indigenous peoples of Latin America and the Caribbean continue to experience political, social, and economic marginalization that has relegated them to conditions of poverty and extreme poverty^67^. This is mainly expressed in the adoption of sectoral laws that subordinate Indigenous rights to business and state interests, and in implementation gaps, particularly in delimitating, demarcating, and titling lands^7^.

Indigenous communities should be seriously considered in national programs to reduce anemia since, as Vázquez et al.^14^ said, anemia prevalence was reduced in Latin American countries only by national programs that covered a wide geographical area, were well monitored, and were extended over time. According to the United Nations Office for the Coordination of Humanitarian Affairs^67^, the purchasing power of Indigenous peoples to access basic commodities, including food, has even been recently decreasing due to quarantine and social mobility limitation measures dictated by governments to contain the SARS-CoV-2 pandemic, thus increasing the risk of food insecurity, particularly in areas where subsistence activities based on traditional land-based livelihoods are not an option. Despite the state responses aimed at containing and mitigating the impact of COVID-19 on Indigenous peoples and the 285 social protection measures adopted in Latin America, the state responses to date have not been tailored to the needs of Indigenous people^67^. In addition, the traditional territories of 108 Indigenous peoples in Latin America straddle national boundaries, which means that cooperation and cohesive strategies between governments are needed to design policies to tackle child anemia as a public health problem.

### Strengths and Limitations of the Study

The main strength of this study is that it is a systematic review, covering a large geographical area where Indigenous populations are common. Also, it recovers data about specific Indigenous communities and national databases. It provides detailed information about the different samples from all the studies about the prevalence of anemia among Indigenous children performed in the last 35 years in Latin America. Nevertheless, it has some limitations. First, we included only articles that explicitly mentioned any Indigenous community in Latin America. Other articles that did not mention that they were studying Indigenous populations or that Indigenous people were part of the overall study sample may have been overlooked. Second, mainly cross-sectional studies were available, which disallows the establishment of causal relationships. Third, as designed, the National Surveys do not provide information about specific Indigenous ethnic groups. Fourth, this systematic review only included data about the prevalence of anemia among Indigenous children. Combining these data with other studies about malnutrition is important to better understand the relationship between the prevalence of anemia and malnutrition in Indigenous children in Latin America. Fifth, limitations in the evaluation of the quality of articles in this systematic review are possible. The STROBE statement was only a guideline for reporting observational studies, not precisely a quality assessment tool. Therefore, we did not exactly assess for the quality of the selected articles, we just gave scores to a description of each selected study.

### Recommendations

We recommend further studies update and extend the overview on anemia as a public health problem in Indigenous communities in Latin America. Besides, studies about the prevalence of anemia among Indigenous children should be combined with other studies about malnutrition to raise more information about how to prevent child anemia. Nutrition programs directed to Indigenous communities should be adjusted to the way of living of these populations without disturbing their cultural traditions. Also, exploring social acceptance, community willingness, and participation of Indigenous populations in nutrition programs is important. Finally, assessing the Indigenous community leaders’ ability to get involved in research and nutrition programs is necessary.

## CONCLUSIONS

Anemia constitutes a severe and poorly documented public health problem among Indigenous children in 21 Indigenous communities in Bolivia, Brazil, Colombia, Costa Rica, Ecuador, Guatemala, Mexico, and Peru. All Indigenous communities included in this study showed child anemia as a public health problem, especially in younger children.
